# Pathways to Highly Oxidized Products in the Δ3-Carene + OH System

**DOI:** 10.1021/acs.est.1c06949

**Published:** 2022-02-04

**Authors:** Emma L. D’Ambro, Noora Hyttinen, Kristian H. Møller, Siddharth Iyer, Rasmus V. Otkjær, David M. Bell, Jiumeng Liu, Felipe D. Lopez-Hilfiker, Siegfried Schobesberger, John E. Shilling, Alla Zelenyuk, Henrik G. Kjaergaard, Joel A. Thornton, Theo Kurtén

**Affiliations:** † Department of Chemistry, University of Washington, Seattle, Washington 98195, United States; ‡ Department of Chemistry, University of Helsinki, Helsinki FI-00014, Finland; § Institute for Atmospheric and Earth System Research (INAR), University of Helsinki, Helsinki FI-00014, Finland; ∥ Department of Chemistry, University of Copenhagen, Copenhagen DK-2100, Denmark; ⊥ Atmospheric Sciences and Global Change Division, Pacific Northwest National Laboratory, Richland, Washington 99354, United States; # Department of Atmospheric Sciences, University of Washington, Seattle, Washington 98195, United States

**Keywords:** atmospheric chemistry, autoxidation, highly oxidized organic molecules (HOMs), monoterpene oxidation, secondary organic aerosol (SOA)

## Abstract

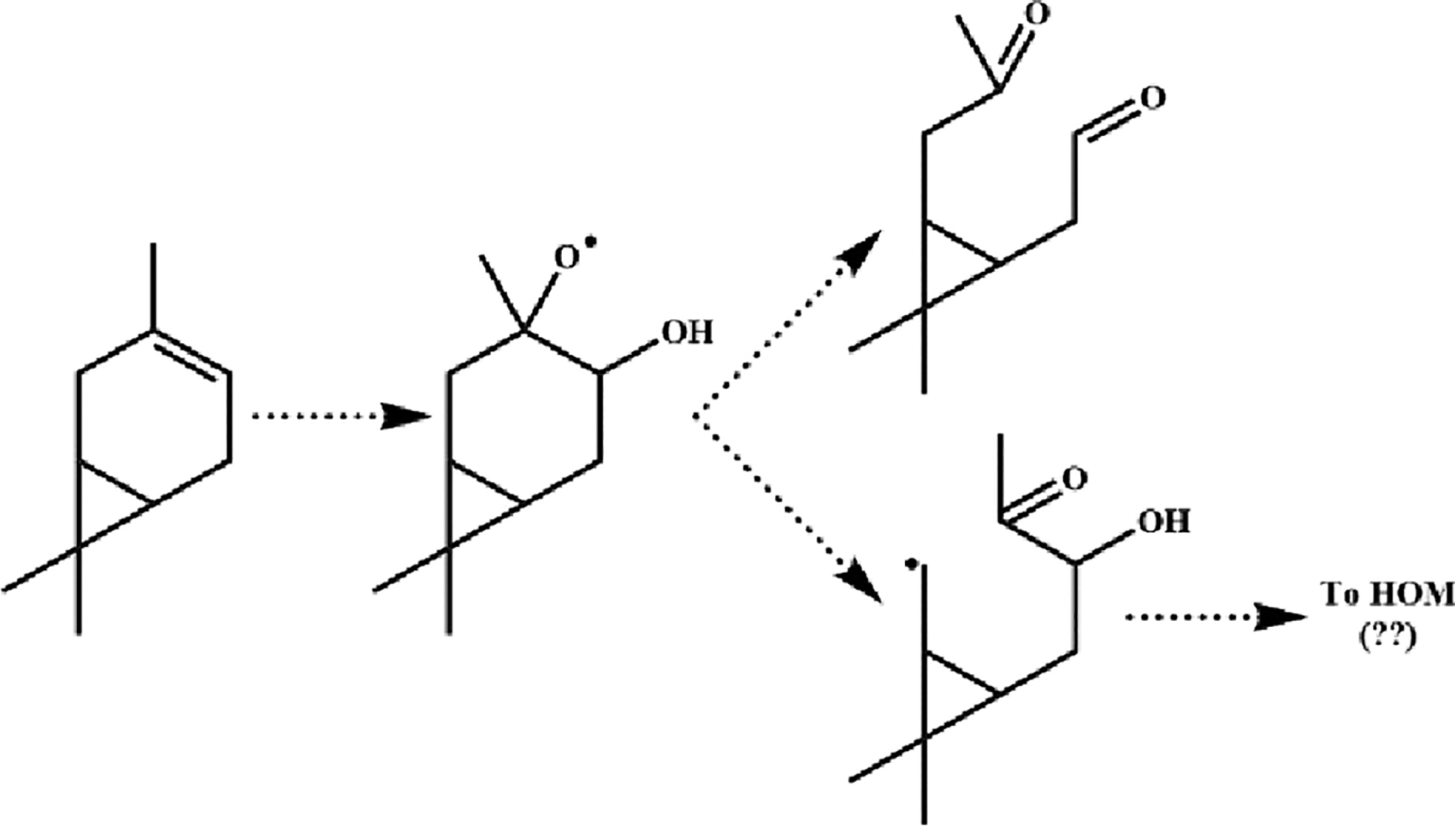

Oxidation of the monoterpene Δ3-carene (C_10_H_16_) is a potentially important and understudied source of atmospheric secondary organic aerosol (SOA). We present chamber-based measurements of speciated gas and particle phases during photochemical oxidation of Δ3-carene. We find evidence of highly oxidized organic molecules (HOMs) in the gas phase and relatively low-volatility SOA dominated by C_7_–C_10_ species. We then use computational methods to develop the first stages of a Δ3-carene photochemical oxidation mechanism and explain some of our measured compositions. We find that alkoxy bond scission of the cyclohexyl ring likely leads to efficient HOM formation, in line with previous studies. We also find a surprising role for the abstraction of primary hydrogens from methyl groups, which has been calculated to be rapid in the α-pinene system, and suggest more research is required to determine if this is more general to other systems and a feature of autoxidation. This work develops a more comprehensive view of Δ3-carene photochemical oxidation products via measurements and lays out a suggested mechanism of oxidation via computationally derived rate coefficients.

## Abstract 2

Laboratory experiments and rate calculations are performed to develop a photochemical oxidation mechanism for Δ3-carene, which may be an important precursor for secondary organic aerosol.

## 1 Introduction

Secondary organic aerosol (SOA), particulate matter that is formed in the atmosphere as opposed to being directly emitted, is a substantial component of submicron aerosols^1,2^ which are particularly detrimental to human health and are the source of much of the uncertainty related to aerosol-climate effects.^3^ One of the largest sources of SOA is the oxidation of biogenic volatile organic compounds (BVOCs)^4^ that are emitted from vegetation and have lifetimes of hours or less against atmospheric oxidation. Monoterpenes (C_10_H_16_), one class of BVOCs, play a well-documented role in SOA formation and growth.

To understand and predict SOA formation and growth, mechanisms of BVOC conversion to lower-volatility compounds are required. One mechanism that has recently garnered much attention is autoxidation.^5^ In the context of atmospheric gas-phase chemistry, autoxidation involves an organic peroxy (RO_2_) or alkoxy (RO) radical abstracting a hydrogen from elsewhere in the molecule, followed by O_2_ addition. Multiple unimolecular steps can rapidly increase the oxygen content of a molecule while keeping the carbon backbone mostly intact.^6^ Autoxidation is thus a pathway to form highly oxygenated organic molecules (HOMs), which are defined to have six or more oxygens.^6^ Most studies on HOM formation and identification have focused on α-pinene, which is considered the most abundant monoterpene.^7^ However, even for the α-pinene ozonolysis system, a molecular-level mechanism for HOM formation was only proposed recently.^8^ Due to the often very low volatility of HOMs, they can impact SOA formation even when formed in low yields; thus, exploring the possibility of other BVOCs to form HOMs is important.

One intriguing monoterpene in this regard is Δ3-carene. Δ3-Carene is predicted to have lower emission rates than α-pinene globally,^7^ although regionally they have been measured in roughly equivalent concentrations.^9^ Additionally, Δ3-carene has a larger OH reaction rate constant (8.0 × 10^–11^ cm^3^ molecule^–1^ s^–1^)^10^ than α-pinene (5.4 × 10^–11^ cm^3^ molecule^–1^ s^–1^).^11^ Taken together, in certain environments such as the boreal forest, OH can react with Δ3-carene as often as α-pinene, suggesting that the production rate of HOM from Δ3-carene photochemical oxidation could be as large as that of α-pinene in these environments, depending on the HOM yields.

Despite the potential importance of Δ3-carene, little is known about its oxidation initiated by OH radicals. Previous Δ3-carene photochemical oxidation studies determined the presence and yield of caronaldehyde (14–77%), a primary first-generation product,^10,12−15^ and an SOA yield of 1.6–40%.^16−19^ However, only one study was carried out in the absence of NO_*x*_^14^ and all the studies used elevated levels of precursors (Δ3-carene and NO_*x*_ when used), suggesting that they likely enhanced the role of bimolecular reactions of radicals over autoxidation compared to the atmosphere.^20^ More recently, 13 compounds with molecular formulas of C_8-10_H_12-16_O_1-4_ were detected in the particle phase, with structures assigned to the formulas based on a combination of the literature and speculation.^21^

We present herein a combined chamber and theoretical study of the OH oxidation of Δ3-carene. We investigate the gas- and particle-phase products of this reaction produced in a steady-state chamber experiment and use known gas-phase organic chemistry to predict possible products matching the molecular formulas we observe. We use quantum chemical calculations to calculate the rate coefficients of various peroxy and alkoxy H-shifts and bond scission/ring opening reactions to predict the most likely unimolecular reactions potentially leading to HOM formation. We develop a mechanism for the first steps of Δ3-carene photochemical oxidation and find that alkoxy bond scission of the cyclohexyl ring is the most likely pathway to HOM formation.

## 2 Methods

### 2.1 Laboratory Experiments

Experiments were performed in the Pacific Northwest National Laboratory (PNNL) environmental chamber^22^ in 2015 as part of the Secondary Organic Aerosol From Forest Emissions Experiment (SOAFFEE), which has been described previously.^20,23,24^ The PNNL chamber is 10.6 m^3^ and was operated in continuous-flow mode with a total flow of 48.2 L min^–1^, resulting in a chamber lifetime of ∼3.7 h. (1*S*)-(+)-3-Carene (90% purity, Sigma-Aldrich, from here on written as Δ3-carene) was passed into the chamber to maintain a steady-state concentration of 10 ppb before lights were switched on to initiate photochemistry. H_2_O_2_ was injected via an automated syringe as a radical OH and HO_2_ precursor at a mixing ratio of 1 ppm H_2_O_2_ before photochemistry was initiated. Quasi-monodisperse 50 nm solid ammonium sulfate seed particles were continually added. The chamber was operated at room temperature and 50% RH.

A suite of online gas and particle-phase instruments were employed to characterize the chemical speciation and concentration within the chamber. Ozone (Thermo Environmental Instruments model 49C), NO/NO_2_/NO_*x*_ (Thermo Environmental Instruments model 42C), and Δ3-carene (proton-transfer-reaction mass spectrometer, Ionicon) concentrations were monitored. Mass loading and bulk submicron particle-phase composition were measured with an Aerodyne high-resolution time-of-flight aerosol mass spectrometer. The chemically speciated gas- and particle-phase compositions of semi- and low-volatility compounds^25^ in near-real time were measured with a high-resolution time-of-flight chemical ionization mass spectrometer (HR-ToF-CIMS) with iodide ionization coupled to a Filter Inlet for Gases and AEROsols (FIGAERO).^26^ We do not convert HR-ToF-CIMS signal to concentration but use the measurements to qualitatively compare compositions and as a basis for building oxidation mechanisms. The operation of the FIGAERO-CIMS has been described previously.^23^ Briefly, the FIGAERO was operated in a cycle with a 43 min aerosol collection and simultaneous real-time gas-phase measurements, followed by a 70 min thermal desorption with a temperature ramp from room temperature to 200 °C at a rate of 10 °C min^–1^, followed by a 10 min cool down to room temperature. Gas-phase zeros were performed by overblowing the pinhole with ultra-high-purity N_2_ every 5 min, and particle-phase blanks were obtained by inserting a secondary filter upstream from the primary collection filter every fourth collection.

In addition, the volatility of size-selected SOA particles was measured using a single-particle mass spectrometer, miniSPLAT, described in detail elsewhere.^27^ Briefly, SOA particles from the PNNL chamber were extracted, size selected with a differential mobility analyzer, passed through two charcoal denuders to remove gas-phase organics, and loaded into a stainless-steel evaporation chamber that was partially filled with activated carbon to continuously remove evaporated organics. The miniSPLAT was used to periodically sample particles from the evaporation chamber to characterize changes in their vacuum aerodynamic diameter and mass spectra. Room-temperature evaporation kinetics of the size-selected SOA particles, expressed as organic volume fraction remaining (VFR) as a function of evaporation time, was quantified by measuring the change in particle vacuum aerodynamic diameter with 0.5% precision, accounting for the volume of the inorganic seed.

### 2.2 Quantum Chemical Rate Calculations

**Scheme 1:**
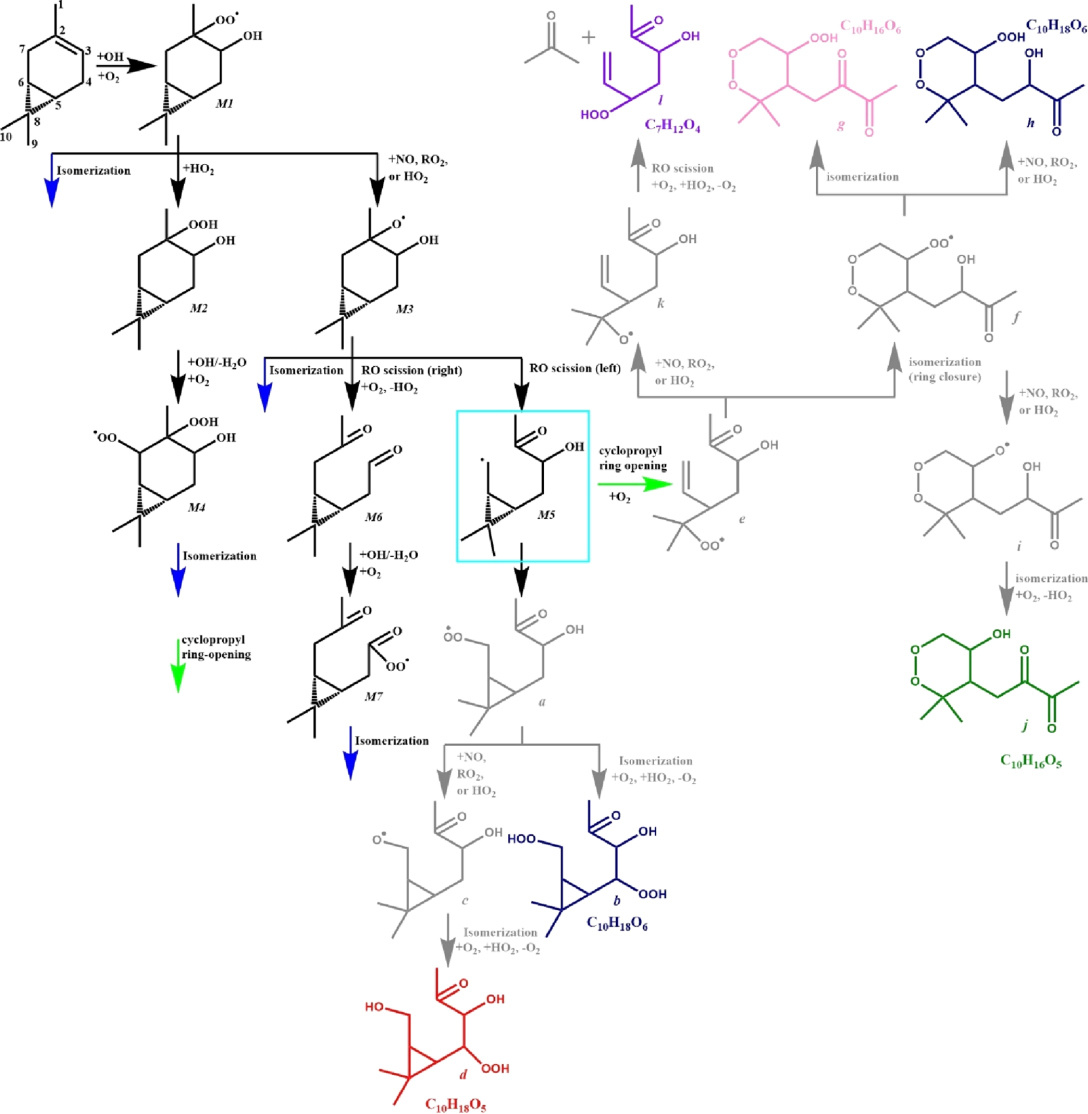
Simplified Mechanism of Δ3-Carene Photochemical Oxidation Pathways studied herein are shown in black with compounds labeled “M#”. Isomerization (H-shift) reactions and cyclopropyl ring-opening reactions that are discussed are highlighted in blue and green, respectively. Carbons are numbered on the parent Δ3-carene for ease of reference in the main text. Speculative autoxidation pathways discussed in Section 3.2.5 are shown via gray and colored structures stemming from M5 and labeled with letters a–j.

Rate coefficient calculations were performed according to the approach in Møller et al.^28^ based on multiconformer transition-state theory (MC-TST)^29^ and presented numerous times before (e.g., refs (30−34)). Briefly, the calculations included six steps: (1) a systematic conformer search was performed for the reactants, products, and transition states in Spartan 16^35^ with the MMFF force field. The FFHINT keyword was utilized to enforce a neutral charge on the radical center, and constraints were applied to selected bond lengths for the transition states. (2) All identified structures were optimized at the B3LYP/6-31+G(d) level of theory in Gaussian 09^36^ or 16.^37^ (3) Unique structures, determined by energy and dipole moments (differences >1 × 10^–5^ H and >1.5 × 10^–2^ D),^38^ within 2 kcal mol^–1^ in electronic energy of the lowest-energy conformer were then optimized at the ωB97X-D/aug-cc-pVTZ level. Additionally, harmonic vibrational frequencies were calculated to obtain zero-point corrected energies of all species and to confirm the character of the optimized transition-state structures with a single imaginary frequency corresponding to the H-shift or bond scission. (4) To obtain a more accurate barrier height, ROHF-ROCCSD(T)-F12a/cc-pVDZ-F12 single-point energy calculations (denoted as “F12” for simplicity from here on) were then performed using Molpro 2015.1^39^ on the lowest-energy ωB97X-D/aug-cc-pVTZ optimized reactant, product, and transition-state conformers for selected reactions of the first-generation alkoxy radical (Scheme 1, M3). Exclusion of F12 singe-point calculations results in a factor of ∼2 average difference in rate coefficients and increases their uncertainty to about 2 orders of magnitude.^40^ (5) Finally, the Eckart tunneling coefficient^41^ was calculated using the forward and reverse barrier heights (energy differences at the ωB97X-D/aug-cc-pVTZ level or at the F12 level where available) between the lowest-energy transition state and the reactant and product conformers connected to it via intrinsic reaction coordinate (IRC) paths at the B3LYP/6-31+G(d) level. The tunneling coefficient calculation also included the imaginary frequency of the transition state at the ωB97X-D/aug-cc-pVTZ level calculated in step 3. (6) Rate coefficients were calculated using MC-TST with the Eckart tunneling coefficients, ωB97X-D/aug-cc-pVTZ partition functions, relative energies between conformers, and zero-point vibrational corrections and electronic energies for the barrier height calculated at either the ωB97X-D/aug-cc-pVTZ or, where available, F12 levels. The pressure dependence of RO_2_ H-shift reactions has been shown to be negligible in the α-pinene ozonolysis system^42^ at 298 K and 1 atm and thus was not studied here as similar results are expected for our reactions.

## 3 Results and Discussion

### 3.1 Δ3-Carene Photochemical Oxidation Products

**Figure 1:**
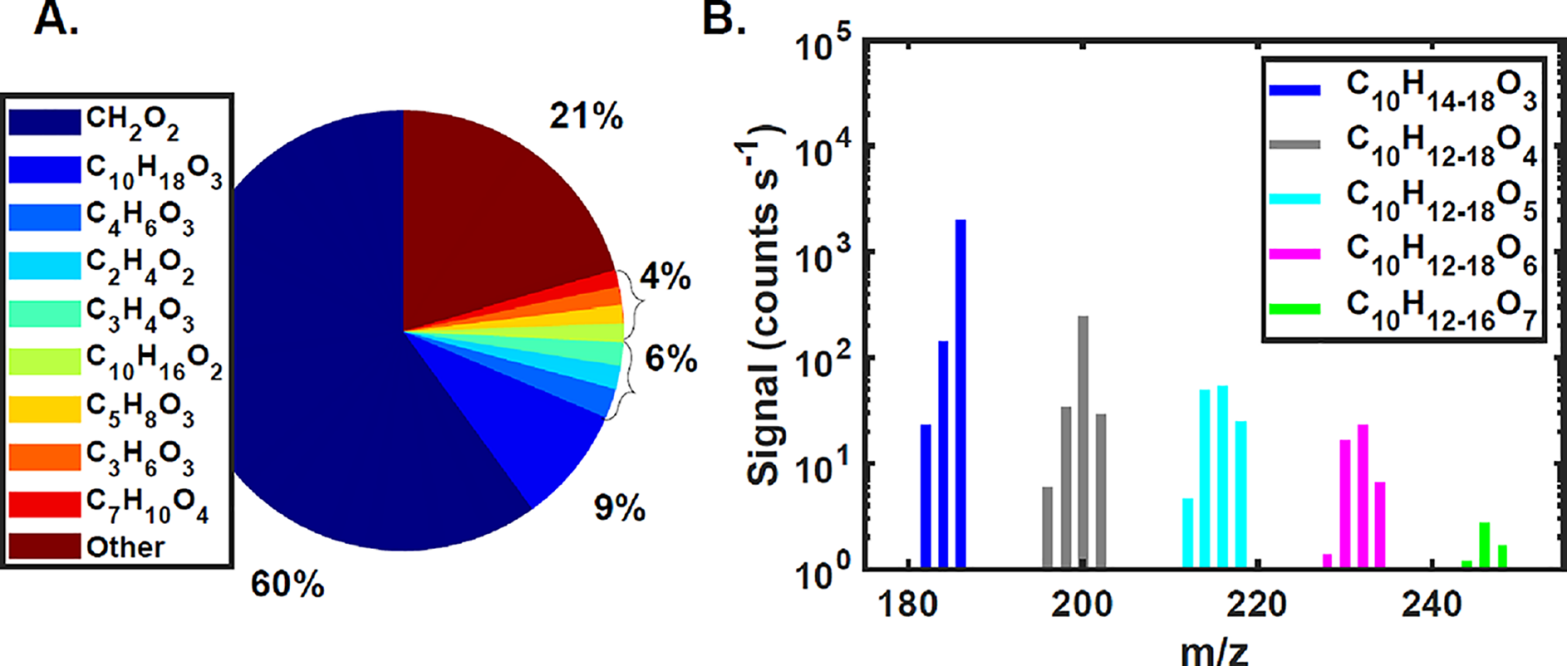
Gas-phase products of Δ3-carene photochemical oxidation. (A) Largest CIMS signals and (B) mass spectrum of C_10_H_12-18_O_3-7_ compounds separated by two hydrogens.

We identified ∼200 organic molecular adducts to iodide ions from Δ3-carene photochemical oxidation using the FIGAERO-CIMS. In the gas phase, the dominant signal is formic acid, CH_2_O_2_ (Figure 1A), consistent with previous work showing that formic acid is a major product.^14^ The next largest signal is C_10_H_18_O_3_, presumably a hydroxy hydroperoxide (Scheme 1, M2) formed via oxidation of the double bond, which is expected given the use of H_2_O_2_ as a radical OH precursor which produces abundant HO_2_.^43^ C_10_H_16_O_2_ is likely caronaldehyde, which was previously shown to form in high yields, 31–77% in the presence of NO_*x*_,^12,13,19^ although much lower (14%) in the absence of NO_*x*_.^14^ Most of the 10 highest abundance gas-phase species by signal have relatively low oxygen numbers and smaller carbon backbones (<C_10_); however, some higher-order oxygenates are detected. Figure 1B shows a series of C_10_H_12-18_O_3-7_ compounds. Higher oxygen numbers are associated with lower gas-phase concentrations, likely due to a combination of lower yields of highly oxygenated compounds and such species being sequestered into the particle phase and chamber walls due to lower vapor pressures and thus stronger partitioning.^44^ We are unaware of other studies reporting Δ3-carene photochemical oxidation products with 10 carbon atoms and more than 4 oxygen atoms.

**Figure 2:**
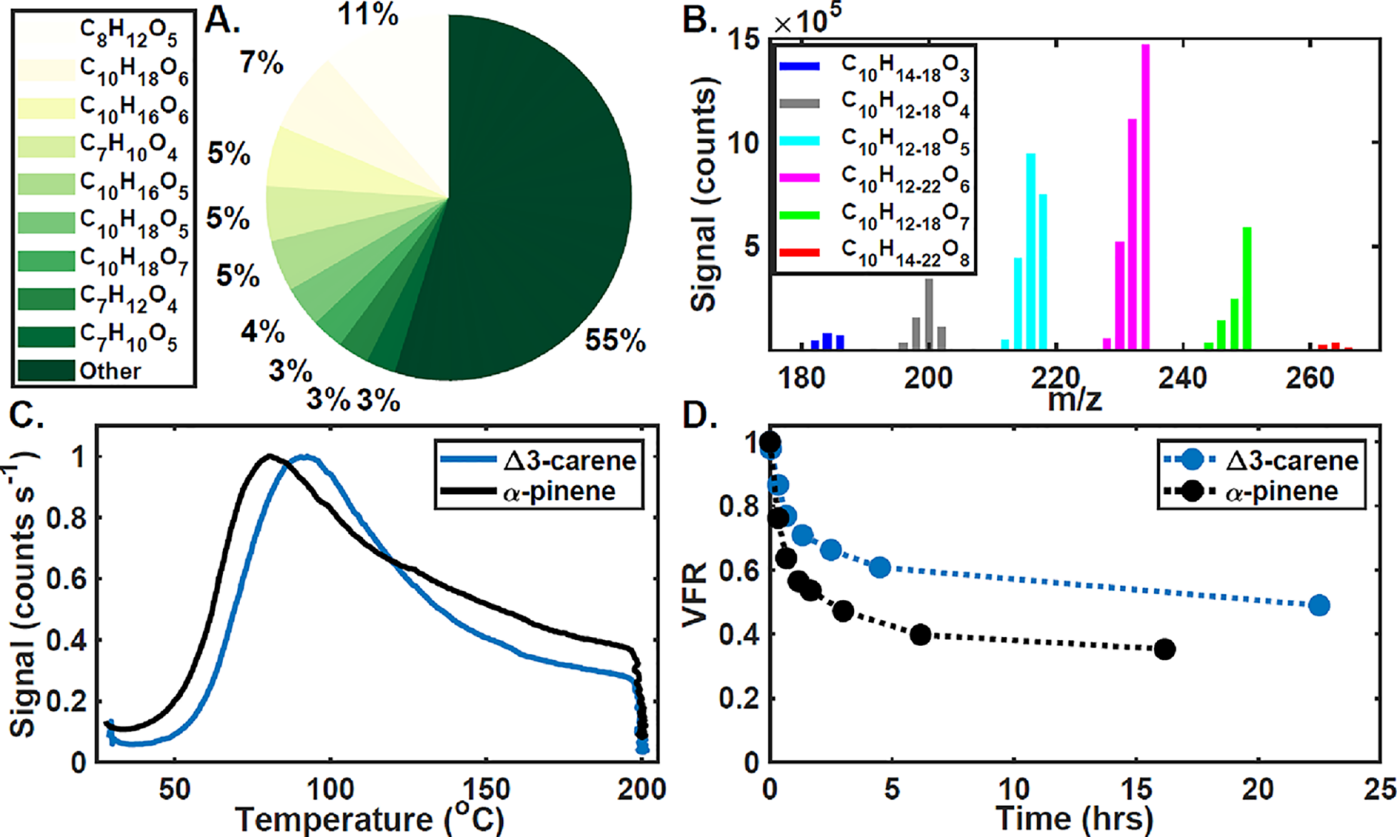
Particle-phase analysis of Δ3-carene photochemical oxidation. (A) Top compounds by the CIMS signal, (B) mass spectrum of C_10_H_12-22_O_3-8_ compounds, separated by two hydrogens, (C) sum thermogram for Δ3-carene (blue) and α-pinene (black), and (D) volume fraction remaining (VFR) for 151 nm particles as a function of evaporation time for Δ3-carene (blue) and α-pinene (black).

Photochemical oxidation of Δ3-carene under the conditions described above produced 4.7 μg m^–3^ of SOA at steady state, corresponding to an SOA mass yield of 31%. The composition of the particle phase (Figure 2A,B) differs from that of the gas phase, as expected. The oxygen content is in general significantly higher in the particle phase, dominated by O_5_ and O_6_ species as opposed to O_2_ and O_3_ species in the gas phase. The detected particle-phase compositions imply the presence of certain reaction mechanisms. For example, C_7_ compounds indicate the efficient loss of a C_3_ group, likely through alkoxy scission after cleavage of the cyclopropyl ring, analogous to the Δ3-carene + NO_3_ system.^45^ It is not obvious how a C_8_ species, the largest molecular signal measured in the particle phase, would be formed from gas-phase chemistry, followed by gas-to-particle partitioning alone. However, C_8_ species also often dominate the particle-phase composition measured with FIGAERO-CIMS in the α-pinene photochemical oxidation system (Lopez-Hilfiker et al.^46^ and Figure S1). The gas- and particle-phase composition of α-pinene photochemical oxidation measured during this campaign is provided in the Supporting Information for comparison.

FIGAERO-CIMS can also provide insight into the volatility of the SOA. We compare the campaign-average thermogram, that is, the average of each individual particle-phase desorption in signal versus temperature space, normalized by maximum signal (Figure 2C), for Δ3-carene relative to the much more studied α-pinene with the same precursor concentrations (10 ppb BVOC, 1 ppm H_2_O_2_). The temperature of maximum desorption (*T*_max_) for the Δ3-carene bulk SOA thermogram is noticeably higher (93 °C) relative to α-pinene (80 °C), indicating that the Δ3-carene SOA is generally of lower volatility.^26^ However, the thermogram for Δ3-carene has a smaller relative contribution from the high-temperature tail and the largest measured signals have large carbon backbones (Figure 2A), indicating the detection of intact molecules formed in the gas phase during the experiment and desorbing directly from the particle phase. On the other hand, α-pinene has a much larger relative contribution of the signal from the high-temperature region and the particle-phase signal is dominated by likely thermal decomposition products (Figure S1A),^47,48^ suggesting decomposition of ELVOC or larger-order structures such as dimers or oligomers. The sum thermogram structures do not definitively indicate volatility however in that the higher SOA concentration in the Δ3-carene system will favor more partitioning of higher-volatility material, which was recently seen for Δ3-carene ozonolysis^49^ and could be the reason for the more distinct peak for Δ3-carene.

To provide an additional constraint on effective volatility, we investigate the room-temperature isothermal evaporative behavior of the two SOA systems and find that Δ3-carene SOA is more recalcitrant toward evaporation than is α-pinene SOA formed under similar conditions (Figure 2C). After 16 h of isothermal evaporation at room temperature,^27^ the VFR for α-pinene SOA is 35%, while after 22.5 h, 49% of the Δ3-carene SOA remains (Figure 2D). These observations together suggest that Δ3-carene SOA has relatively lower volatility than α-pinene SOA. Given these results, we conclude that Δ3-carene represents a potentially important contribution to ambient SOA. Thus, we explore the initial stages of oxidation to develop an oxidation mechanism and identify potential oxidation products.

### 3.2 Δ3-Carene Photochemical Oxidation Mechanism

A gas-phase mechanism of Δ3-carene photochemical oxidation in the presence of NO_*x*_^50^ was developed based on laboratory experiments,^16,17^ but to our knowledge, no multistep mechanism exists for photochemical oxidation in the absence of NO_*x*_. Here, we use the measured compositions from the previous section and the current understanding of atmospheric chemistry of organic radicals to propose a mechanism for Δ3-carene + OH in a NO_*x*_-free environment via autoxidation (Scheme 1). We start with an OH addition to the double bond, resulting in a carbon-centered radical on the tertiary carbon, previously shown to be the major addition channel.^51^ While OH could also add to the other side of the double bond or abstract a hydrogen,^51^ we focus on the most likely reaction here and at each subsequent stage. After OH addition, O_2_ will add at the radical site, resulting in a peroxy radical with four possible stereoisomers, depending on the side of the ring reacting (Scheme 1, M1).^34^ We then assume three fates of this peroxy radical: (1) H-shift reactions (isomerization), (2) termination via HO_2_, dominant in the chamber experiments described above (Scheme 1, M2), or (3) bimolecular reaction with HO_2_, RO_2_, or NO, yielding an alkoxy radical (Scheme 1, M3). M3 could also be formed via photolysis of the closed-shell M2. From here, we calculate the rate coefficients of different likely reactions of these first-generation radicals and further reactions of two second-generation products that are likely to be formed in high yields. Each of these pathways is described in detail below and shown in Scheme 1.

#### 3.2.1 First-Generation RO_2_ H-Shifts

The first-generation RO_2_ (Scheme 1, M1) can undergo two types of bimolecular reactions. The first is termination via HO_2_ to a hydroperoxide (Scheme 1, M2) which will not condense to aerosol due to the relatively high saturation vapor concentration (1.9 × 10^3^ to 9 × 10^3^ μg m^–3^, depending on the stereoisomer, as estimated with COSMO*therm*^52^ and as described in Kurtén et al.^53^). However, being a likely high-yield product in our chamber (the second largest composition by signal, Figure 1A), we perform further calculations on this termination product, discussed in Section 3.2.4. Reaction with HO_2_ in our chamber or NO or RO_2_ in the atmosphere could also lead to an alkoxy radical (Scheme 1, M3). Alkoxy radicals are reactive, and we discuss the fate of this molecule in depth in the next section.

We investigate three H-shifts for the peroxy radical (Scheme 1, M1): from the α carbon with the −OH group (1,4), from the −OH group on the α carbon (1,5), or from the methyl group on the three-membered ring (1,7). However, none of these H-shift reactions are likely to be competitive with bimolecular reactions in our laboratory experiments or under any atmospheric conditions, as has been shown previously.^34^ The calculated highest rate coefficients of the possible stereoisomers are 5.5 × 10^–5^ s^–1^ for the 1,4 shift, 1.7 × 10^–3^ s^–1^ for the 1,5 shift, and 2.6 × 10^–6^ s^–1^ for the 1,7 shift (Table S1). The corresponding H-shifts were also calculated to be slow (<10^–4^ s^–1^) in the Δ3-carene + NO_3_ system.^54^

The product of the H-shift from the α carbon will be a ketone, terminating this pathway and not leading to a HOM or otherwise low-volatility product. The H-shift from the −OH group produces a hydroperoxy alkoxy radical which has the potential to break open the ring, but the formation of this alkoxy is not likely. Neither of these products is investigated further. The fate of the carbon-centered radical on the methyl group attached to the cyclopropyl ring will not be explored further either as its formation is also unlikely, although the fate of a similar carbon-centered radical is discussed in Section 3.2.3 and can be used as a framework for the fate of this radical.

#### 3.2.2 Fate of the First-Generation Alkoxy Radical

The hydroxy alkoxy radical (Scheme 1, M3), formed from the bimolecular reaction of the first-generation RO_2_ with HO_2_, NO, or RO_2_, is an intriguing precursor for HOMs and low-volatility product formation. The alkoxy alcohol has two likely fates: breaking the six-membered ring, resulting in M5 and M6, or abstracting a hydrogen from one of the methyl groups on the three-membered ring, which is sterically accessible to the alkoxy radical in the 3D structure (Figure S2). The cyclohexyl ring can break either toward C7, the CH_2_ group labeled “7” on Δ3-carene in Scheme 1, resulting in M5, or toward C3, the CH–OH group labeled “3” on Δ3-carene in Scheme 1, resulting in M6 after abstraction of the alcohol’s hydrogen via O_2_. The branching will favor breaking toward C3 due to the stabilization from the −OH electron-withdrawing group, leading to caronaldehyde (Scheme 1, M6),^55^ which we further investigate in Section 3.2.4 due to the potentially high yields. However, we calculate that both reactions have large rate constants (left: 1.1 × 10^7^ to 2.1 × 10^9^ s^–1^, right: 1.3 × 10^9^ to 1.2 × 10^10^ s^–1^) with low barrier heights (left: 4.6–8.2 kcal mol^–1^, right: 4.0–5.5 kcal mol^–1^) for each stereoisomer (Table 1), indicating that the ring break toward C7 can occur in competition with ring break toward C3. If the ring breaks toward C7, this could either lead to HOMs by O_2_ addition and further H-shift reactions (see Section 3.2.5 and Scheme 1) or lead to the breaking of the cyclopropyl ring (see the next section), which has been shown to be important for HOM formation in the Δ3-carene + NO_3_ system.^45,54^ Even low yields of HOMs can impact SOA formation due to their often very low volatility, implying that small fractions breaking toward C7 are still atmospherically relevant. Because the ring breaking toward C7 and C3 are close in rates and barrier heights, F12 single-point corrections are included in the alkoxy ring breaking rate coefficient calculations to increase precision.

**Table 1:**
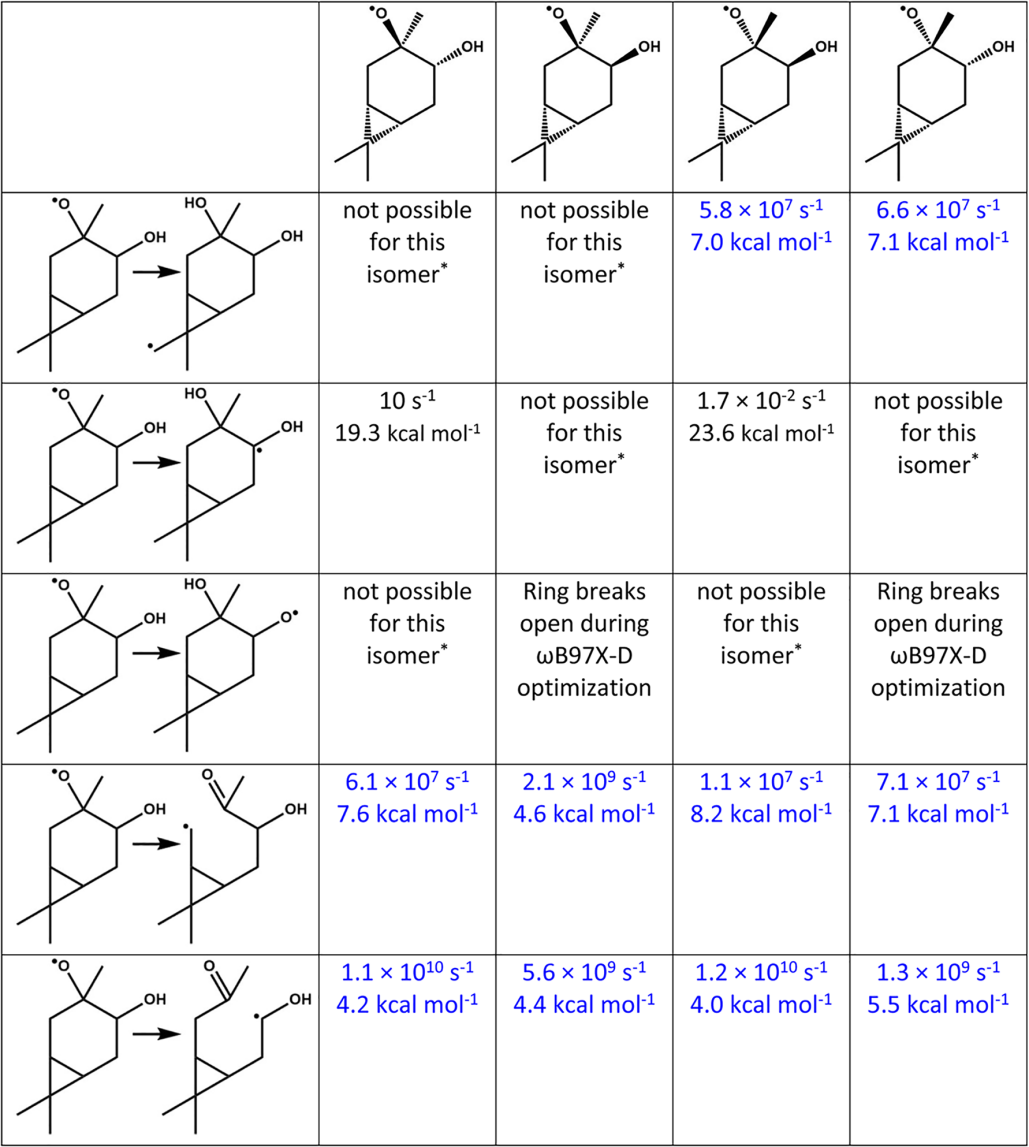
Fate of the First-Generation Alkoxy Radical M3 from Scheme 1^a^

a Rate coefficients (units of s^–1^) and forward barriers (units of kcal mol^–1^) are shown for each stereoisomer. Blue values indicate the inclusion of F12 single-point corrections, and black values are calculated with barrier heights at the ωB97X-D/aug-cc-pVTZ level.

* Steric hindrance prevents this reaction from occurring in the isomer in question.

The alkoxy radical can also perform H-shifts. We investigated three different H-shifts (Table 1) and found that only the shift from one of the methyl groups on the propyl ring could be competitive with ring breaking. This shift will be approximately as likely as the ring breaking toward C7, with rate coefficients of 5.8 × 10^7^ and 6.6 × 10^7^ s^–1^ for the two stereoisomers where this shift is geometrically available. It is generally assumed that H-abstractions from primary carbons are slow,^56^ although recent findings suggest that for specific systems, they may be competitive.^57^ We therefore include F12 single-point energy calculations in these H-shifts as well to compare with the alkoxy ring breaking as accurately as possible. We explore the fate of the resulting primary carbon-centered radical in the next section. The other possible H-shifts are predicted to be much slower than the methyl H-shifts and the ring breaking reactions, so these do not include F12 single-point calculations. Table 1 details the rate constants and barrier heights for each of these reactions.

#### 3.2.3 Cyclopropyl Ring Opening

As described above, opening of the cyclopropyl ring could be an important pathway to HOM formation,^45,54^ so we studied this reaction in greater depth (Table S2). The primary carbon-centered radical α to the cyclopropyl ring, be it formed via H-abstraction from the methyl group (Table S2, reactant in the first two rows) or from the breaking of the cyclohexyl ring (Table S2, reactant in the bottom row), has two main fates. The first is addition of an O_2_ to form a peroxy radical, assumed to occur at ∼10^7^ to 10^8^ s^–1^ under atmospheric conditions.^11,58,59^ In competition with this bimolecular reaction, the radical may also be able to break open the cyclopropyl ring, forming a double bond and a more stable secondary or tertiary carbon-centered radical. We calculated the rate constants for the cyclopropyl ring openings and found them to be fast, on the order of 10^7^ to 10^8^ s^–1^ (Table S2). Thus, we anticipate contributions from both O_2_ addition and ring opening. We expect these products to be good candidates to form HOMs as breaking the cyclopropyl ring will increase the flexibility and therefore the ability to perform further H-shifts after O_2_ addition, particularly in the case where the cyclohexyl ring is also already broken (Table S2, bottom row). Furthermore, the double bond that is generated in each of the three cases will be reactive to OH addition, leading to further oxidation with the potential for additional H-shifts or peroxy ring closing reactions and a lowering of the volatility.^34^

#### 3.2.4 Fate of Second-Generation Products

HOM formation from second-generation products will not be prompt due to the inherent time required to form the reactant, that is, the first-generation product, but their isomerization rates will typically be enhanced due to the presence of multiple functional groups^5,32^ and multigeneration SOA formation is well documented.^60−66^ As stated above, caronaldehyde (Scheme 1, M6) is a first-generation product with widely varying observed yields (14–77%)^12−15^ and measured to be 1% of the total gas-phase signal here (Figure 1A), although the signal does not equate to the overall concentration and iodide CIMS is not expected to be especially sensitive to caronaldehyde.^25^ Due to its potentially high yields, we investigate its fate further. The most likely reaction pathway for caronaldehyde is the abstraction of the aldehyde hydrogen via OH,^61,67,68^ followed by O_2_ addition, yielding a second-generation RO_2_ (Scheme 1, M7). This RO_2_ can then undergo reaction with HO_2_ to form a carboxylic acid^69−71^ or a peroxy acid, both of which are estimated to have relatively high vapor pressures (carboxylic acid: 1.2 × 10^3^ μg m^–3^, peroxy acid: 50 μg m^–3^^52^), or the RO_2_ can undergo an H-shift. We investigated a 1,7 H-shift from the carbon α to the ketone and found that it is not likely to compete with bimolecular reaction as the calculated rate constant is 2.4–2.9 × 10^–4^ s^–1^ (Table S3). We conclude that this pathway likely does not lead to HOM formation.

The other second-generation product we investigate is the hydroxy hydroperoxide (Scheme 1, M2, and Table S4), which is the second largest gas-phase signal measured (9%, Figure 1A) in our chamber. One option for further oxidation of this molecule is an H-abstraction from the molecule via OH. The most likely H-abstraction is from C3^61,67,68^ or the −OH group on C3, although this will likely terminate in a ketone. To investigate a possible second-generation H-shift, we assume an H-abstraction from C7 (Scheme 1), followed by O_2_ addition to form M4, and an H-shift from C3, leading to the formation of a ketone. We calculate the rate coefficient of the H-shift for each of the four possible peroxy stereoisomers and find them to be slow (0.6–36 × 10^–9^ s^–1^) and therefore unlikely (Table S4). This is meant to be a representative test of peroxy radical H-shifts stemming from M2. Peroxy radicals located at different positions on the molecule may have faster H-shifts, although it is likely that they will still abstract the hydrogen from C3 as it is the most acidic,^61,67,68^ terminating the molecule as a ketone that will be of a relatively high volatility (9–110 μg m^–3^, depending on the stereoisomer^52^). The second-generation peroxy radical (Scheme 1, M4) could also abstract the hydrogen off the −OOH or −OH group, which could lead to further unimolecular reactions and potential oxygenation, although we do not explore these possibilities here.

#### 3.2.5 Potential Reactions

Figures 1A and 2A show the molecular compositions of species measured in the gas and particle phases, respectively. The only overlap with our computationally developed mechanism (Scheme 1) is C_10_H_18_O_3_, presumably a hydroxy hydroperoxide, and C_10_H_16_O_2_, presumably caronaldehyde. Additionally, in Section 3.2.2, we speculated based on previous work^45,54^ that breaking the cyclohexyl ring toward C7 via an alkoxy (Scheme 1, M3) is a likely path toward HOM formation. To fill the gap between measured and modeled species, we present possible reactions leading to several of the measured compositions in the particle phase (Scheme 1, gray and colored compounds), some of which are HOMs (i.e., containing six or more oxygens), from the carbon-centered radical intermediate (Scheme 1, M5). Throughout this discussion, we do not address all possible reaction pathways and products but simply the most likely or those that lead to measured compositions via autoxidation. We note that biomolecular reactions likely produce many of the same molecular compositions discussed in this section, although we do not explore them herein. We also do not show every possible H-shift location for a molecule but choose one for illustration. Many of these reactions are analogous to those probed in Scheme 1 of Draper et al.,^45^ where NO_3_ was the oxidant rather than OH, leading to slightly different functionalization.

There are two direct fates of the carbon-centered radical (Scheme 1, M5). First, O_2_ could add to the carbon-centered radical, forming a peroxy radical (a), which can then terminate as C_10_H_18_O_6_ (b, navy) or C_10_H_18_O_5_ (d, red). Second, the carbon-centered radical (Scheme 1, M5) could rearrange, opening the cyclopropyl ring as discussed in Section 3.2.3 and Table S2, followed by O_2_ addition, leading to a peroxy radical (e). The peroxy radical (e) could perform a ring closure + O_2_ addition (f) and then terminate to another isomer of C_10_H_18_O_6_ (h, navy), perform an H-shift to form C_10_H_16_O_6_ (g, pink), or become an alkoxy which then performs an H-shift to generate C_10_H_16_O_5_ (j, green). Finally, the peroxy radical (e) could become an alkoxy (k), which could lose acetone to form C_7_H_12_O_4_ (l, purple). In all, we predict five molecular compositions from this one radical intermediate (Scheme 1, M5).

A few overarching themes, along with some further questions, arise from the speculative mechanism. First, we find multiple possible isomers of C_10_H_18_O_6_ (Scheme 1, b and h), a reminder that each molecular formula measured likely contains multiple isomers, possibly with varying I^–^ CIMS sensitivity. Second, we find that the O_5_ species (d and j) are generated from alkoxy radicals (c and i), while we predict that the C_7_ (l), of which there are 3 in the top 10 measured particle-phase signals (Figure 2A), is the result of cleavage of the cyclopropyl ring followed by alkoxy scission to remove three carbons. Both the O_5_ and O_7_ pathways we propose are expected to be enhanced with increasing NO_*x*_ concentrations because they both stem from alkoxy radicals.

We note that our mechanistic speculations are based on the assumption that products measured in the particle phase are produced in the gas phase, which may not necessarily be true. For example, C_7_H_12_O_4_ is the eighth most abundant signal in the particle phase (Figure 2A) but is expected to have a relatively high volatility: the structure predicted in Scheme 1 is predicted to have a saturation vapor concentration of 340–470 μg m^–3^, depending on the stereoisomer.^52^ Instead of forming in the gas phase and then condensing, C_7_H_12_O_4_ could be formed via thermal decomposition of a different particle-phase species during thermal desorption, as shown previously.^47,48,72^ However, for C_7_H_12_O_4_, the thermogram does not show typical signs of thermal decomposition (Figure S3).

Similar to α-pinene, some Δ3-carene products retain the same degree of unsaturation (DOU) as the parent compound while simultaneously becoming more oxygenated and lower volatility. Δ3-Carene has three DOUs (two rings and one double bond), as do, for example, C_10_H_16_O_*n*_ and C_7_H_10_O_*n*_ species, and C_8_H_12_O_5_, the largest signal measured in the particle phase (Figure 2A). This pattern is most likely to occur via carbonyl functionalities and in some cases the formation of endoperoxides which are important in other BVOC oxidation systems.^8,73^ Three structural examples are the dihydroperoxy ketone that is likely to form from the second-generation H-shift of the hydroxy hydroperoxide discussed in Section 3.2.4 or molecules g and j in Scheme 1. However, the addition of endoperoxides or ketones is not expected to lower the volatility substantially: the predicted vapor concentration of molecule g in Scheme 1 is 80–140 μg m^–3^, while the ketone formed from the H-shift in Table S4 and discussed in the previous section is 9–110 μg m^–3^, depending on the stereoisomer.^52^ If instead the carbonyls were part of carboxylic or peroxy acid groups, the vapor pressure would likely be much lower.^74^

## 4 Atmospheric Implications

We found that the first-generation RO_2_ (Scheme 1, M1) is most likely to form an alkoxy radical (Scheme 1, M3) in competition with terminating channels of bimolecular reaction. These alkoxy radicals will predominantly break the cyclohexyl ring toward C3, the carbon to which OH initially adds, and form caronaldehyde (Scheme 1, M6). However, we calculate that a non-negligible fraction will ring-break toward C7, a CH_2_ group (Scheme 1, M5), from which it is easier to develop plausible mechanisms for HOM formation (Scheme S1). Additionally, some of these alkoxy radicals are likely to undergo H-shifts from the methyl group on the cyclopropyl ring, which could lead to opening of the cyclopropyl ring and likely to HOMs as well as to seven-carbon products detected in the chamber study. If the first-generation RO_2_ terminates in a hydroxy hydroperoxide (Scheme 1, M2), it can undergo H-abstraction via OH, although the resulting molecule will not likely undergo H-shifts and is instead expected to terminate in a dihydroperoxy ketone. The RO_2_ resulting from the OH oxidation via H-abstraction from caronaldehyde (Scheme 1, M7) is also unlikely to undergo H-shifts. Therefore, both second-generation RO_2_ are most likely to terminate with HO_2_ to form ROOHs. The RO_2_ could however also form RO, which are more likely to lead to HOMs, although RO formation is less likely in the case of caronaldehyde where the RO would be an acyl RO that is likely to decompose and lose CO_2_. In light of these considerations, we conclude that the main pathway for forming HOMs from OH oxidation of Δ3-carene will likely be from alkoxy ring breaking toward C7 (Scheme 1, M5).

An interesting finding of this work is the surprisingly rapid abstraction of primary H’s from methyl groups by alkoxy radicals, which is generally assumed to be slow,^56^ but has been calculated to be rapid in the α-pinene system.^57^ More work is needed to investigate the possibility of these types of isomerization reactions in other BVOCs, particularly monoterpenes that tend to have multiple methyl groups. Additionally, mechanisms describing how a molecule can retain the DOU of the parent compound while being oxidized to products of sufficiently low volatility to partition to the particle phase, for example, C_10_H_16_O_6_, are still unclear. HOMs with multiple DOU have been observed in the gas phase,^8,75^ but the predicted structures are typically multifunctional bicyclic or contain carboxylic acids,^75^ which we do not predict in Scheme 1.

## Supporting Information Available

α-Pinene photochemical oxidation products, volatility of a Δ3-carene photochemical oxidation product, a 3D compound, and rate coefficients and barrier heights for reactions in section 3.2 (PDF)
