# Supplementary material for: Pathways to Highly Oxidized Products in the Δ3-Carene + OH System
Source: Environ Sci Technol. 2022 Feb 4;56(4):2213–24. doi: 10.1021/acs.est.1c06949 (PMC8956127; doi:10.1021/acs.est.1c06949)
Supplement: Supplementary file 1 — es1c06949_si_001.pdf [file es1c06949_si_001.pdf]

Supporting Information for:

Pathways to highly oxidized products in the Δ3-carene + OH system

Emma L. D'Ambro[1,2,!], Noora Hyttinen[2,3,@], Kristian H. Møller[4], Siddharth Iyer[2,3,#], Rasmus V. Otkjær[4], David M. Bell[5,$], Jiumeng Liu[5,%], Felipe D. Lopez-Hilfiker[6,^], Siegfried Schobesberger[6,&], John E. Shilling[5], Alla Zelenyuk[5], Henrik G. Kjaergaard[4], Joel A. Thornton[1,6*], Theo Kurtén[2,3*]*

[1]Department of Chemistry, University of Washington, Seattle, WA, 98195, USA
[2]Department of Chemistry, University of Helsinki, Helsinki, FI-00100, Finland
[3] Institute for Atmospheric and Earth System Research (INAR), University of Helsinki, Helsinki, FI-00100, Finland
[4]Department of Chemistry, University of Copenhagen, Copenhagen, DK-2100, Denmark
[5]Atmospheric Sciences and Global Change Division, Pacific Northwest National Laboratory, Richland, WA, 99354, USA
[6]Department of Atmospheric Sciences, University of Washington, Seattle, WA, 98195 USA

Present Addresses
[!]Present address: Office of Research and Development, US Environmental Protection Agency, Research Triangle Park, NC, 27711, USA
[@]Present address: Department of Chemistry, Nanoscience Center, University of Jyväskylä, Jyväskylä, FI-40014, Finland
[#]Present address: Aerosol Physics Laboratory, Tampere University, Tampere, FI-33014, Finland
[$]Present address: Laboratory of Atmospheric Chemistry, Paul Scherrer Institute, Villigen, CH-5232, Switzerland
[%]Present address: School of Environment, Harbin Institute of Technology, Harbin, Heilongjiang, 150001, China
[^]Present address: Tofwerk AG, Thun, CH-3645, Switzerland
[&]Present address: Department of Applied Physics, University of Eastern Finland, Kuopio, FI-70210, Finland

Author Information

Corresponding Authors

[*]Email: dambro.emma@epa.gov
[*]Email: thornton@atmos.uw.edu
[*]Email: theo.kurten@helsinki.fi

This file includes:

Pages S1 – S8
Figures S1 – S3
Tables S1 – S4
References

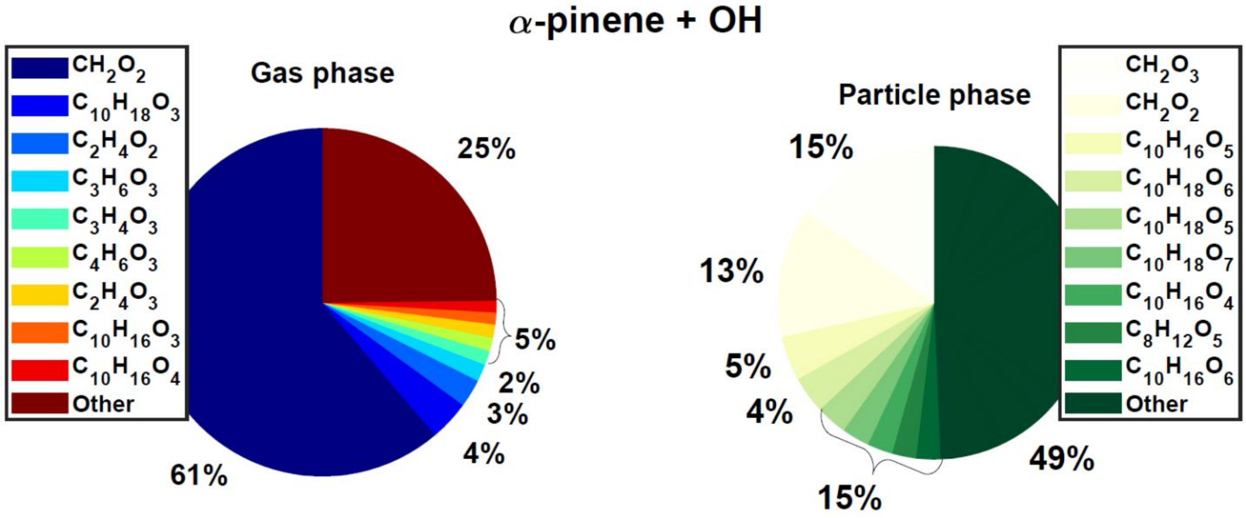

Figure S1. Top gas- and particle-phase signals for α-pinene photochemical oxidation under similar conditions (10 ppb VOC, 1 ppm H_2O_2, 50 nm ammonium sulfate seed) in the same chamber.

The top gas- and particle-phase signals from α-pinene photochemical oxidation are shown in Figure S1 to compare with Figure 1A and Figure 2A. In the gas phase, CH_2O_2, presumably formic acid, also makes up ~2/3 of the total signal for α-pinene, as it does for Δ3-carene. Recall that the signal is uncalibrated so does not necessarily reflect concentrations in the chamber. The next most abundant signal is also $C_{10}H_{18}O_3$ for α-pinene as it is for Δ3-carene, again likely a hydroxy hydroperoxy as a result of relatively high HO_2 concentrations[1]. From there, the compositions differ slightly between the two BVOC, but are not dramatically different in carbon chain length or degree of oxidation. The similarity in structure is not entirely surprising considering the starting composition is the same with slight structural differences.

On the other hand, the particle phase for α-pinene looks markedly different than Δ3-carene. When oxidized under similar conditions (10 ppb VOC, 1 ppm H_2O_2, 50 nm ammonium sulfate seed) in the same chamber, 2.1 μg m^{-3} of SOA was produced at steady state, relative to 4.7 μg m^{-3} in the case of Δ3-carene (main text). α-Pinene is dominated by 1-carbon species, indicating an important role for thermal decomposition[2, 3]. However, the remainder of the top 10 species by signal predominantly have the carbon backbone intact, while Δ3-carene has several C_7 and C_8 species in the top 10. Notably $C_8H_{12}O_5$ is in both the Δ3-carene and α-pinene top 10. Many of the molecular formulas are similar between the two BVOC though, particularly $C_{10}H_{16-18}O_{4-7}$ species.

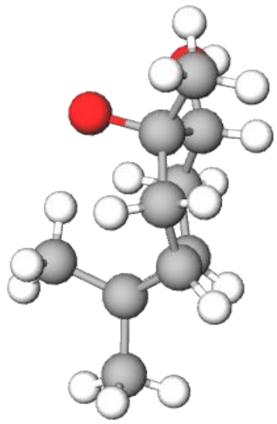

Figure S2. First generation alkoxy alcohol (*M3* from Scheme 1 in the main text) that can abstract a hydrogen from the methyl group on the three-membered ring.

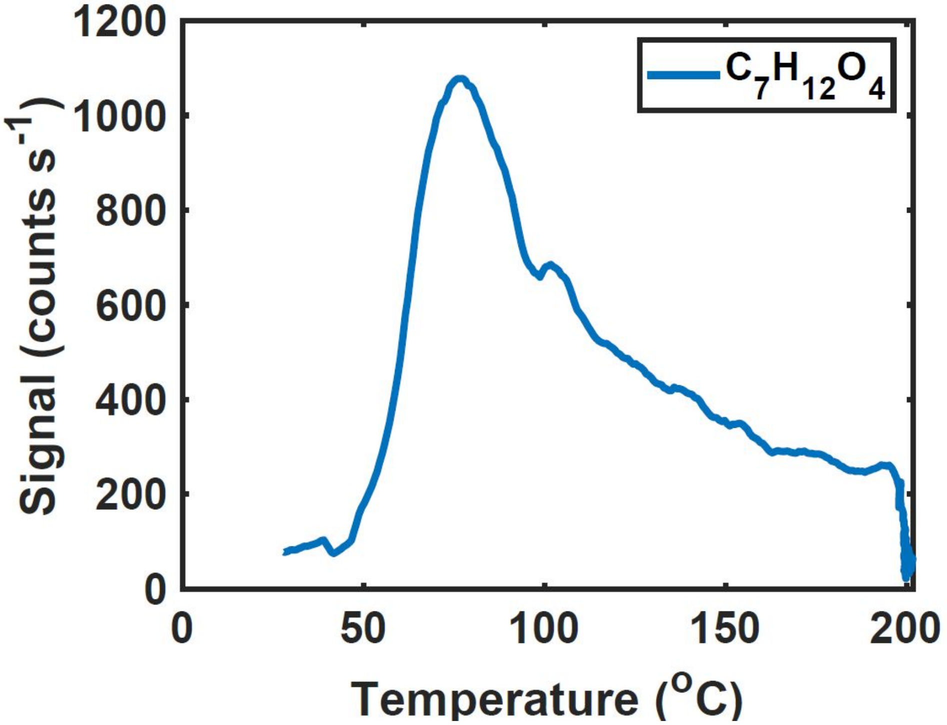

Figure S3. Thermogram of $C_7H_{12}O_4$.

Table S1. Isomerization reactions of the first generation peroxy radical (*M1* from Scheme 1 in the main text). Rate coefficients (units of s^{-1}) and forward barriers (units of kcal mol^{-1}) are shown for each stereoisomer at the ωB97X-D/aug-cc-pVTZ level.

	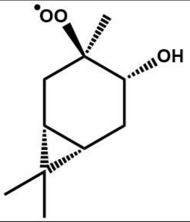	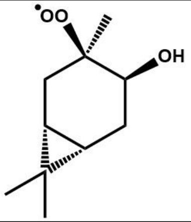	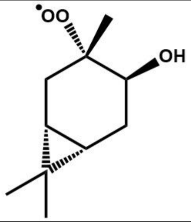	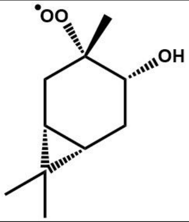
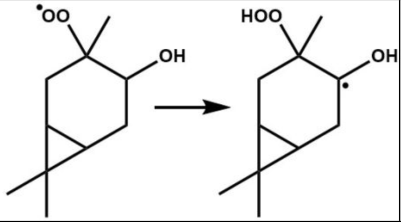	5.5×10^{-5} s^{-1}<br>26.6 kcal mol^{-1}	8.7×10^{-6} s^{-1}<br>28.2 kcal mol^{-1}	5.8×10^{-6} s^{-1}<br>27.4 kcal mol^{-1}	2.4×10^{-9} s^{-1}<br>32.4 kcal mol^{-1}
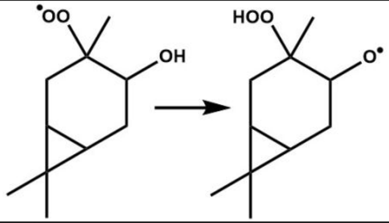	3.1×10^{-5} s^{-1}<br>23.0 kcal mol^{-1}	9.8×10^{-4} s^{-1}<br>21.5 kcal mol^{-1}	2.6×10^{-4} s^{-1}<br>21.4 kcal mol^{-1}	1.7×10^{-3} s^{-1}<br>20.4 kcal mol^{-1}
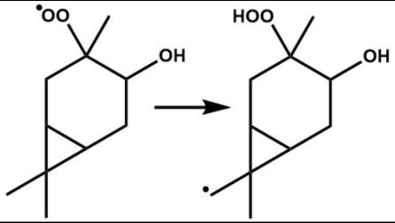	not possible for this isomer*	not possible for this isomer*	4.5×10^{-13} s^{-1}<br>38.7 kcal mol^{-1}	2.6×10^{-6} s^{-1}<br>26.4 kcal mol^{-1}

*steric hindrance prevents this reaction from occurring in the isomer in question

Table S2. MC-TST rate coefficients (unit of s^{-1}) and forward barrier heights (unit of kcal mol^{-1}) for cyclopropyl ring openings at the ωB97X-D/aug-cc-pVTZ level. The precursor in the first two rows is the product of isomerization of *M3* in Scheme 1, and the precursor in row 3 is *M5*.

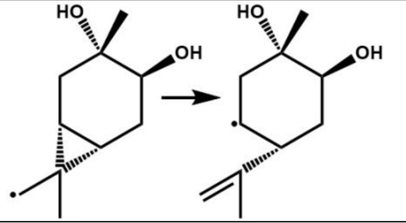	9.5×10^{7} s^{-1}<br>6.7 kcal mol^{-1}
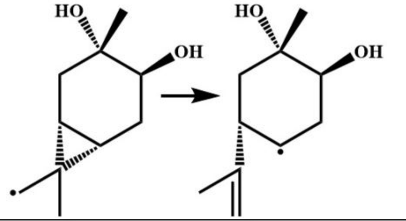	1.1×10^{7} s^{-1}<br>8.1 kcal mol^{-1}
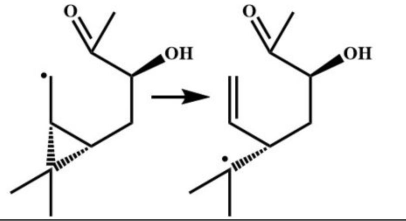	1.9×10^{8} s^{-1}<br>6.0 kcal mol^{-1}

Table S3. Potential H-shift reaction for different stereoisomers of caronaldehyde (*M6* from Scheme 1 in the main text) photochemical oxidation. Rate coefficients (units of s^{-1}) and forward barriers (units of kcal mol^{-1}) are shown for each stereoisomer at the ωB97X-D/aug-cc-pVTZ level.

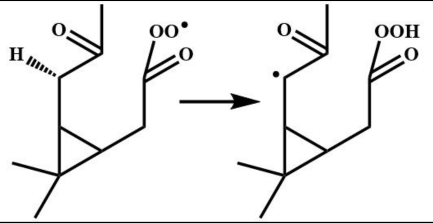	2.9×10^{-4} s^{-1}<br>23.6 kcal mol^{-1}
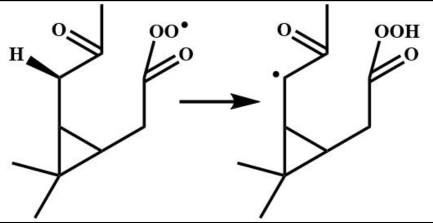	2.4×10^{-4} s^{-1}<br>23.6 kcal mol^{-1}

Table S4. Potential H-shift reactions from the photochemical oxidation of the first-generation hydroxy hydroperoxide. Rate coefficients (units of s^{-1}) and forward barriers (units of kcal mol^{-1}) are shown for each stereoisomer at the ωB97X-D/aug-cc-pVTZ level.

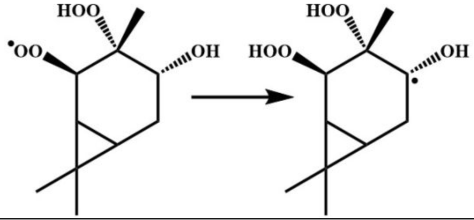	5.9×10^{-10} s^{-1} 30.5 kcal mol^{-1}
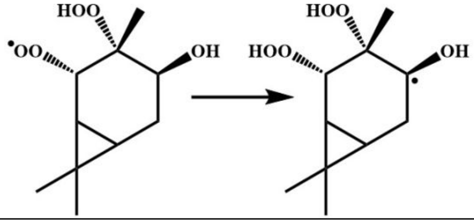	6.1×10^{-9} s^{-1} 28.4 kcal mol^{-1}
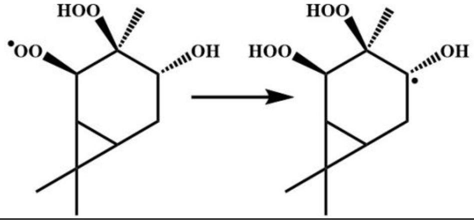	3.6×10^{-8} s^{-1} 25.5 kcal mol^{-1}
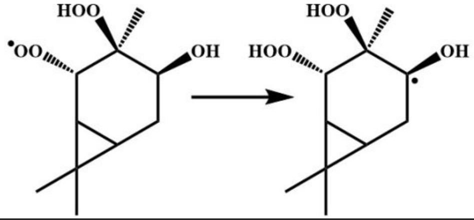	1.2×10^{-9} s^{-1} 28.9 kcal mol^{-1}

References

1. D'Ambro, E. L.; Møller, K. H.; Lopez-Hilfiker, F. D.; Schobesberger, S.; Liu, J. M.; Shilling, J. E.; Lee, B.; Kjaergaard, H. G.; Thornton, J. A., Isomerization of Second-Generation Isoprene Peroxy Radicals: Epoxide Formation and Implications for Secondary Organic Aerosol Yields. *Environ. Sci. Technol.* **2017,** *51* (9), 4978-4987.
2. Lopez-Hilfiker, F. D.; Mohr, C.; D'Ambro, E. L.; Lutz, A.; Riedel, T. P.; Gaston, C. J.; Iyer, S.; Zhang, Z.; Gold, A.; Surratt, J. D.; Lee, B. H.; Kurten, T.; Hu, W. W.; Jimenez, J.; Hallquist, M.; Thornton, J. A., Molecular composition and volatility of organic aerosol in the southeastern US: Implications for IEPOX derived SOA. *Environ. Sci. Technol.* **2016,** *50* (5), 2200-2209.
3. Schobesberger, S.; D'Ambro, E. L.; Lopez-Hilfiker, F. D.; Mohr, C.; Thornton, J. A., A model framework to retrieve thermodynamic and kinetic properties of organic aerosol from composition-resolved thermal desorption measurements. *Atmos. Chem. Phys.* **2018,** *18* (20), 14757-14785.
